# Quality of Life Among Saudi Parents of Children With Attention-Deficit/Hyperactivity Disorder: A Cross-Sectional Study

**DOI:** 10.7759/cureus.63911

**Published:** 2024-07-05

**Authors:** Mohammad A Jareebi, Ahmad Y Alqassim, Ibrahim M Gosadi, Mousa Zaala, Ramis Manni, Taif Zogel, Emtnan Robidiy, Fatimah Qarn, Shatha Moharaq, Wafa Alharbi, Aisha Alhobani, Mostafa Mohrag

**Affiliations:** 1 Family and Community Medicine Department, Jazan University, Jazan, SAU; 2 Family and Community Medicine Department, Faculty of Medicine, Jazan University, Jazan, SAU; 3 Psychiatry, King Khalid University, Abha, SAU; 4 Faculty of Medicine, Jazan University, Jazan, SAU

**Keywords:** family well-being, saudi arabia, child behavior, parental quality of life, adhd

## Abstract

Background

Attention-deficit/hyperactivity disorder (ADHD) in children can adversely impact parental quality of life (QoL). However, limited research exists among families in Saudi Arabia, especially in understudied regions like Jazan. This study was designed to determine the QoL in parents of children with ADHD in Jazan, Saudi Arabia.

Methodology

This cross-sectional study was conducted in the Jazan region of Saudi Arabia from June 2023 to December 2023. Online questionnaires were used to collect data from parents of children with ADHD residing in the country. A structured questionnaire was developed in consultation with the National Institute for Children’s Health Quality (NICHQ) guidelines. The total sample size was 276. The study participants were recruited through convenient non-random snowballing sampling where the promotion of the questionnaire web link aided in reaching the required sample size of the study. Participants aged more than 18 years, residents of the Jazan area, and both genders were included. R software was used for data analysis.

Results

The study, involving 275 participants, revealed that 45 (16%) children were diagnosed with ADHD by doctors, with a higher prevalence among males 36 (80%). The NICHQ scale identified ADHD in 50 (18%) participants, with the predominant subtypes being inattentive 28 (56%), hyperactive/impulsive 28 (56%), and combined 19 (38%). The median QoL score was 8.6, with good QoL reported by 51.27% of parents and poor QoL by 48.73%. Gender significantly influenced QoL (p < 0.01), with 57% of males and 43% of females reporting good QoL, while 61% of females and 39% of males reported poor QoL. Oppositional defiant symptoms were significantly associated with poorer QoL (p < 0.05). There was a notable alignment between ADHD diagnoses from parental reports and the NICHQ scale.

Conclusions

This cross-sectional study found that parents of children with ADHD behaviors, especially those exhibiting oppositional defiant symptoms, experienced significantly lower QoL, particularly in personal fulfillment and psychosocial well-being. The findings emphasize the need for culturally tailored psychosocial interventions in Saudi Arabia to improve parental coping and QoL, benefiting overall family well-being and child outcomes.

## Introduction

Attention-deficit/hyperactivity disorder (ADHD) is a common neurodevelopmental condition affecting children worldwide, with prevalence estimates ranging from 2.2% to 7.2% [1]. Beyond its impact on children, ADHD poses a substantial burden on families, academic and occupational functioning, and public health due to its high financial costs and adverse effects on self-esteem [2,3]. This disorder is characterized by debilitating behavioral challenges and has been identified as a key contributor to disease burden among youth [2,3]. The repercussions of ADHD extend to parental quality of life (QoL) and family dynamics. Parents of children with ADHD often experience heightened stress, communication difficulties, and problematic parent-child interactions [3]. Additionally, ADHD is associated with increased risks of accidental injuries, substance abuse, academic underachievement, and career difficulties later in life. Collectively, these issues can decrease health-related quality of life (HRQoL) for both affected children and their caregivers [4].

HRQoL is an important focus in healthcare practice and research concerning children with ADHD, as it encompasses an individual’s functioning and subjective well-being across physical, mental, and social health domains [5-7]. From a social-ecological perspective, the interconnectedness of family members recognizes the potential impact of raising children with ADHD on parental well-being. The prevalence of ADHD is higher among males than females, with symptoms presenting as inattentiveness, hyperactivity, or a combination. Although biological diagnostic markers remain elusive, the assessment of ADHD employs a multidimensional approach, including clinical interviews, observations, and parent/teacher ratings. Treatment strategies include pharmacological interventions (e.g., stimulants), behavioral therapies, and cognitive training [8].

Previous studies reveal associations between parenting stress and the severity of child ADHD symptoms, behavioral issues, and maternal mental health conditions. Lower socioeconomic status has also been linked to poorer physical QoL in parents [9-13]. These findings highlight the complex relationship between pediatric ADHD and diminished parental well-being. Comprehensive support measures are needed for families managing this disorder. In Saudi Arabia, ADHD prevalence among school-aged children ranges from 0.7% to 16.4% [14-16]. However, limited research exists on parental QoL and well-being, especially in understudied regions like Jazan. Earlier studies in major cities found higher stress and disrupted family relationships among parents of children with ADHD versus normally developing children [17,18]. The perspectives of rural parents remain overlooked.

Therefore, the main aim of this study is to investigate the QoL of parents who had children with ADHD in the rural Jazan region. The findings will provide insights into the individual, family, and sociocultural factors influencing parental well-being, informing supportive interventions tailored to families in underserved communities.

## Materials and methods

Study design and participants

This cross-sectional study was conducted in the Jazan region of Saudi Arabia from June 2023 to December 2023. Online questionnaires were used to collect data from parents of children with ADHD residing in the country. Informed consent was obtained from all participants. The study protocol adhered to the ethical principles of the Declaration of Helsinki.

The authors obtained information about the diagnosed ADHD cases through various sources, such as medical records, healthcare databases, or previous studies. In addition, if the cases mentioned were undiagnosed, it was crucial to clarify their status and refrain from labeling them as ADHD cases without clinical confirmation. This distinction was important to ensure the accuracy and reliability of the study’s findings.

The target population comprised adult residents of the Jazan region in southwest Saudi Arabia who were parents of children with ADHD. Participants were recruited online through social media platforms, local community forums, and healthcare providers. The surveys were designed to gather information anonymously, ensuring the confidentiality of participants. All participants aged more than 18 years, residents of the Jazan area, and both genders were included, Individuals under 18 years old or not residing in Jazan were excluded to maintain the focus on the specific demographic being studied.

Sample size calculation

A sampling of the study participants was done through convenient non-random snowballing sampling where the promotion of the questionnaire web link likely aided in reaching the required sample size of the study. The sample size estimation of the current study was based on an expected prevalence of family dysfunction of 79.2% which was retrieved from the study by Azazy et al. which measured QoL and family functioning among Egyptian parents of children affected with ADHD [13]. Using the StatCal function of Epi Info, assuming a prevalence of 79.2%, 95% confidence interval, and a 5% acceptable margin of error, a sample of 276 parents was estimated to meet the objective of the current investigation.

Data collection tool and measures

A structured questionnaire was developed in consultation with the National Institute for Children’s Health Quality (NICHQ) guidelines. It contained three sections, namely, demographics, ADHD diagnosis, and QoL assessment.

Demographic information collected included child’s age and gender, parents’ education, employment status, income, residence, and marital status. Other relevant variables were also assessed as potential confounders of parental QoL, including financial problems, psychiatric disorders, health issues, and family psychiatric history.

The validated 10-item Arabic version of the Multicultural Quality of Life Index (MQLI) was utilized to evaluate QoL. This tool measures physical, mental, social, and functional well-being on a 10-point scale from “bad” (1) to “outstanding” (10). The final score is the average across the 10 items, ranging from 10 to 100.

The Vanderbilt Parent Rating Scale was used to screen for ADHD, which contains 55 items mapping onto the Diagnostic and Statistical Manual of Mental Disorders, Fifth Edition diagnostic criteria. One parent completed this for each child participant aged 6-12.

Pilot testing

The questionnaire was pilot-tested on 10 mothers and 10 fathers to assess clarity, suitability, and completion time. Minor modifications were made based on feedback before finalization. Pilot data were not included in the analysis.

Data analysis

Data analysis utilized R software (version 4.3.2), focusing on exploring relationships among sociodemographic characteristics, ADHD-related variables, and the dependent variable, QoL. The initial analysis involved a comprehensive review of overall sample characteristics. Descriptive statistics, including mean, standard deviation, median, and interquartile range (IQR), were computed for continuous variables such as the age of the father and mother, body mass index, and the number of family members. Categorical variables, including gender, educational attainment, income, and residence, were summarized through frequency distributions. To assess the dependence between categorical variables and QoL, the chi-squared test was employed. Furthermore, a two-sample t-test and analysis of variance were utilized to evaluate mean differences among numeric QoL scores. Statistical significance was determined with a threshold p-value below 0.05.

## Results

Sociodemographic characteristics

The study included 275 participants. The mean age was 43 ± 9.4 years for fathers and 37 ± 8.1 years for mothers. Households had an average of 3.5 ± 1.9 children. The sample comprised 139 (51%) males and 136 (49%) females. Regarding education, 115 (42%) fathers and 119 (43%) mothers completed secondary school or below. A bachelor’s degree was held by 140 (51%) fathers and 149 (54%) mothers. Postgraduate education was attained by 20 (7%) fathers and seven (3%) mothers. Most fathers were employed (n = 229, 83%), while 93 (34%) mothers were employed. Annual household income ranged from less than 5,000 SAR (n = 19, 7%) to ≥15,000 SAR (n = 82, 30%) (Table 1).

**Table 1:** Sociodemographic characteristics of the sample (n = 275). SD: standard deviation; n: sample size

Characteristics	Mean ± SD
Father’s age	43 ± 9.4 years
Mother’s age	37 ± 8.1 years
Number of children per household	3.5 ± 1.9 child
Characteristics	Frequency (%)
Gender	
Male	139 (51%)
Female	136 (49%)
Father education	
Secondary school and below	115 (42%)
Bachelor	140 (51%)
Postgraduate	20 (7%)
Father employment	
Employed	229 (83%)
Not employed	46 (17%)
Mother education	
Secondary school and below	119 (43%)
Bachelor	149 (54%)
Postgraduate	7 (3%)
Mother employment	
Employed	93 (34%)
Not employed	182 (66%)
Income	
Less than 5,000	19 (7%)
5,000–9,999	67 (24%)
10,000–14,999	107 (39%)
≥15,000	82 (30%)
Residence	
Rural	156 (57%)
Urban	119 (43%)
Sufficient income perceived by participants	
Yes	157 (57%)
No	118 (43%)

Attention-deficit/hyperactivity disorder characteristics

The mean age of children with ADHD was 7.3 ± 4.0 years. Abnormal behaviors were first noticed by parents at 4.5 ± 2.3 years on average. ADHD was diagnosed by a doctor for 45 (16%) children. Five (2%) families had multiple children with ADHD, while 270 (98%) had only one affected child. Among diagnosed cases, 36 (80%) were male and nine (20%) were female. ADHD impacted parental QoL for 29 (64%) children (Table 2).

**Table 2:** Characteristics of children with ADHD. ADHD: attention-deficit/hyperactivity disorder

Characteristics	Frequency (%)
ADHD	
Yes	45 (16%)
No	230 (84%)
Multiple children with ADHD in the family?	
Yes	5 (2%)
No	270 (98%)
Gender of children with ADHD (n = 45)	
Male	36 (80%)
Female	9 (20%)
Does your ADHD child affect your quality of life? (n = 45)	
Yes	29 (64%)
No	16 (36%)

Attention-deficit/hyperactivity disorder diagnosis and subtypes

Based on the NICHQ scale, 50 (18%) screened positive for ADHD. The predominant subtypes were inattentive (n = 28, 56%), hyperactive/impulsive (n = 28, 56%), and combined (n=19, 38%). Oppositional defiant symptoms were present in 30 (60%), while 19 (38%) had conduct-related issues. Anxiety/depression symptoms occurred in 20 (40%) (Table 3).

**Table 3:** ADHD diagnosis and subtypes (n = 50). ADHD: attention-deficit/hyperactivity disorder; NICHQ: National Institute for Children’s Health Quality

Characteristics	Frequency (%)
ADHD (based on the NICHQ scale)	50 (18%)
Subtypes	
Inattentive	28 (56%)
Hyperactive	28 (56%)
Inattentive + hyperactive	19 (38%)
Oppositional defiant screen	30 (60%)
Conduct screen	19 (38%)
Anxiety/Depression screen	20 (40%)

Parental quality of life

The mean QoL score in the sample was 7.8 ± 2.3. Due to the non-normal distribution of the QoL variable, the median and IQR were employed to characterize the central tendency. The median QoL score was 8.6, and the IQR was 2.6. To dichotomize the scores, the median (8.6) was used to distinguish between individuals with good or poor QoL. Parents reporting good QoL comprised 141 (51.27%), while those reporting poor QoL constituted 134 (48.73%) of the sample.

Factors impacting parental quality of life

Gender significantly influenced QoL (p < 0.01), with 57% of males versus 43% of females reporting good QoL, while 39% of males versus 61% of females reporting poor QoL. No associations were found between QoL and other sociodemographic factors such as age, education, employment, income, or residence. Among ADHD variables, oppositional defiant symptoms were significantly associated with poorer QoL (p < 0.05). No relationships were observed between QoL and factors such as the child’s age, onset age, diagnosis status, gender, or other ADHD subtypes (Table 4).

**Table 4:** Association of quality of life across study variables. ADHD: attention-deficit/hyperactivity disorder; NICHQ: National Institute for Children’s Health Quality; SD: standard deviation

	Quality of life				
Variable	Good		Poor		P-value
	Mean	SD	Mean	SD	
Father’s age	43	8.6	43	11	0.427
Mother’s age	37	8	37	8.5	0.325
Number of children	3.5	1.8	3.4	1.9	0.632
Gender					
Female	43%		61%		<0.01
Male	57%		39%		
Father education					
Bachelor	49%		54%		0.245
Postgraduate	9%		4%		
Secondary school and below	42%		42%		
Father employment					
Employed	84%		82%		0.098
Not employed	16%		18%		
Mother education					
Bachelor	55%		52%		0.068
Postgraduate	3%		1%		
Secondary school and below	41%		47%		
Mother employment					
Employed	32%		36%		0.325
Not employed	68%		64%		
Income					
10,000–15,000	42%		33%		0.351
5,000–10,000	22%		29%		
Less than 5,000	7%		7%		
More than 15,000	30%		30%		
Residence					
City	46%		38%		0.698
Village	54%		62%		
Sufficient income					
No	45%		39%		0.357
Yes	55%		61%		
Child’s age with ADHD	7	4.3	7.7	3.6	0.785
ADHD onset suspected	4.4	2.3	4.9	2.4	0.685
ADHD (diagnosed by a doctor)	17%		15%		0.986
Multiple children with ADHD	2%		1%		0.322
Gender of children with ADHD					
Female	18%		36%		<0.01
Male	82%		64%		
ADHD (based on the NICHQ scale)	16%		22%		0.887
ADHD (inattentive)	11%		8%		0.355
ADHD (hyperactive)	10%		10%		0.695
ADHD (combined: inattentive + hyperactive)	7%		7%		0.221
ADHD (oppositional defiant screen)	8%		17%		0.021
ADHD (conduct screen)	4%		11%		0.087
ADHD (anxiety/depression screen)	6%		10%		0.0716

Alignment of parent-reported and screening diagnoses

Our investigation revealed a remarkable alignment between ADHD diagnoses obtained through parental reports and those derived from the NICHQ scale. For ADHD diagnosed by doctors, 45 (16%) participants confirmed a positive diagnosis, while 230 (84%) did not. Correspondingly, the NICHQ scale identified ADHD in 50 (18%) participants, with 225 (82%) showing no indication of the condition.

## Discussion

This study in the Jazan region of Saudi Arabia found that parents of children with ADHD symptoms experienced poorer QoL compared to parents of children without ADHD. The most notable findings were the lower scores on the Index among parents of ADHD children across the domains of personal fulfillment and psychological well-being. The research aimed to explore the potential impact of diminished parental QoL on the well-being of their children. The study assessed various facets of life, including occupational, interpersonal, social-emotional, personal fulfillment, and physical and psychological well-being. These key measures provided insights into the self-reported QoL of parents raising children with ADHD symptoms.

The findings revealed that parents of children with ADHD experienced diminished QoL across various domains. This is consistent with past research showing that the pressures of parenting a child with ADHD can negatively impact parental physical health, psychological well-being, social relationships, and occupational functioning [10,13,19]. A key finding was the significant association between impaired parental QoL and adverse effects on their children’s well-being. This aligns with the family systems perspective which recognizes the interconnectedness between parental and child outcomes [7]. When parents experience chronic stress, depression, social isolation, and role dysfunction due to caring for a child with ADHD, it can disrupt parenting behaviors and the home environment [20-22]. These disruptions, in turn, exacerbate symptoms, functional impairments, and QoL deficits in the affected child. There is a cyclical, bidirectional relationship between parental QoL and child adjustment. Targeting improvements in parental well-being through supportive interventions can facilitate more optimal developmental, health, and QoL outcomes for children with ADHD.

The finding of diminished parental QoL associated with having a child with ADHD aligns with past research such as the study by Cappe et al. [11] which also reported lower QoL among parents of children with ADHD. Possible reasons suggested for this negative impact were increased caregiving burden and chronic distress in parenting a child with ADHD [10,11]. However, unlike Cappe et al., our study found that mothers and fathers were both affected in terms of parental QoL, whereas they reported a greater impact on mothers. Further, the domains of psychosocial well-being and personal fulfillment were most impaired in our sample, contrasting with the greater physical QoL deficits seen in the past study [11]. The current study also identified oppositional and conduct problems as factors exacerbating parental QoL issues, extending current knowledge on potential mediators [23]. Differences across studies may relate to cultural variations in coping and family dynamics influencing parental QoL [24,25]. Overall, our findings add to the evidence of ADHD-related challenges faced by parents across settings.

Regarding ADHD subtypes, our study found the inattentive subtype to be most prevalent at 9.9%, followed by the hyperactive/impulsive subtype at 8.7%, and, lastly, the combined type at 6.5%. This pattern contrasts with the systematic review by Alhraiwil et al. [26] on ADHD epidemiology in Arab countries, where the hyperactive subtype was more common (1.4%-7.8%) compared to the inattentive subtype (2.1%-2.7%). The differences may relate to variations in community versus clinical samples and assessment methods used across studies [27,28]. Our findings add to the knowledge of ADHD subtype profiles, but more research is needed on potential factors influencing inattentive presentations in our setting, and subsequent impacts on parental QoL.

Contrary to expectations, parental employment status was not significantly associated with QoL, except for employed fathers. This contrasts with the study by Nath et al. [29] in Bangladesh that found employment status impacted QoL for both parents. The reasons for this discrepancy are unclear but may involve cultural differences in perceived gender roles and family responsibilities [21,24]. Surprisingly, education level also showed no relationship with parental QoL unlike past research [30]. A potential explanation could be that awareness of ADHD itself does not necessarily mitigate the stresses of parenting a child with behavioral issues if adequate professional and social support is lacking [10,31]. Overall, our non-significant findings indicate that having a child with ADHD can negatively impact parental QoL regardless of sociodemographic factors. These findings underscore the need for a multidimensional approach addressing psychosocial, systemic, and contextual factors influencing parental well-being.

Unlike the study by Azazy et al. [13] which found lower QoL among urban parents of ADHD children, our study found no significant association between living place and parental QoL. A potential reason is that in our setting, stigma around mental health issues and lack of ADHD awareness may be prevalent across both urban and rural areas [32]. Rural families also face challenges such as limited professional support and access barriers that could negatively impact QoL similar to urban parents [33]. Surprisingly, family income also did not affect parental QoL, contrasting with past research [13]. This suggests financial pressures may not be the main QoL determinant for parents of ADHD children in our context compared to emotional, social, and parenting stresses [10]. More research is needed to elucidate the complex individual, family, and community factors influencing parental QoL in both urban and rural regions to inform contextualized support.

The study revealed a higher prevalence of ADHD among boys, aligning with numerous pervasive research studies suggesting a greater frequency of ADHD among boys. Western studies have consistently reported a higher association of ADHD with male sex [34-37]. In Qatar, the incidence of ADHD symptoms among boys was noted to be 14.1%, whereas among girls, it was 4.4% [38]. This observation is consistent with the common trend that ADHD is more frequently diagnosed in boys than in girls, a pattern observed in many childhood-onset behavioral disorders.

The higher prevalence of ADHD among boys aligns with numerous studies showing a male preponderance, with male-to-female ratios ranging from 2:1 to 9:1 [34-37]. This pattern is consistently observed across cultures. Several factors may contribute to the gender difference. Biologically, sex hormones are proposed to play a role, as testosterone influences neurotransmitter systems implicated in ADHD [39]. Boys may also exhibit more externalizing symptoms associated with hyperactivity-impulsivity, leading to higher clinical referrals [40]. Socially, greater expectations for self-control and quiet behavior in girls could mask inattentive symptoms [41]. Gender norms emphasizing activity in boys may pathologize normal exuberance [42]. Overall, the reasons for the male dominance in ADHD prevalence are likely multidimensional, involving biological susceptibilities interacting with sociocultural influences on symptom expression and identification. More research is needed on female manifestations of ADHD and potential gender biases in diagnosis.

Two notable factors this study identified as influencing parental QoL were family history of psychiatric disorders and chronic parental illness. The adverse impact of parental mental health issues is supported by past research showing higher stress and poorer coping in parents with conditions such as depression and anxiety [22]. Possible mechanisms include genetic susceptibility leading to less optimal parenting, modeling of negative cognitive styles, and diminished social support [43]. Regarding chronic physical illness, the added burden of managing a complex condition could compound the parenting challenges and emotional toll of having a child with ADHD [44]. There may also be direct biological effects of medical illness that disrupt parental neuropsychiatric functioning [45]. These factors underscore the need for a biopsychosocial approach considering familial psychopathology and medical factors that could exacerbate QoL reductions in parents of children with ADHD.

This study provides initial data on the relationship between ADHD symptomatology in children and parental QoL in Saudi Arabia, given the lack of prior regional research on this topic. Findings from other sociocultural contexts may not apply fully to Saudi families’ experiences. Our results lay the groundwork for future investigations to build context-specific knowledge surrounding parental well-being and its linkages to pediatric ADHD in Saudi healthcare settings. Larger-scale quantitative studies assessing generalizability along with qualitative research elucidating parents’ lived experiences are warranted. This can inform culturally tailored interventions to support Saudi families managing ADHD and enhance parental QoL. This study represents an early step toward addressing the evidence gap regarding the influences of pediatric ADHD on parental well-being within the unique social fabric of Saudi Arabia.

This cross-sectional study has limitations warranting cautious interpretation. The self-report survey methodology is subject to response biases. The design shows association, not causation. Online questionnaires prevented gauging participants’ reactions and verifying the accuracy of responses. Mothers comprised most respondents, potentially limiting generalizability to fathers’ perspectives. The non-random sampling approach may have led to self-selection biases. Children’s treatments were not assessed, which can influence ADHD symptoms and parental stress. The sample was restricted to one region, and may not represent wider populations. Reliance on ADHD screening rather than confirmed clinical diagnosis has implications for diagnostic accuracy. Some factors such as family dynamics were not measured. Future longitudinal studies with objective clinical data, diverse participants, multi-region samples, and comprehensive measurements can extend the findings. This exploratory study provides preliminary evidence on ADHD and parental QoL to inform more rigorous investigations.

## Conclusions

This study found that parents of children exhibiting ADHD behaviors experienced substantially lower QoL compared to parents of children without ADHD, especially in domains of personal fulfillment and psychosocial well-being. Oppositional defiant symptoms in the child significantly worsened parental QoL which aligned with the bidirectional relationship between the child and parental well-being. Unique patterns were seen including a high prevalence of the inattentive ADHD subtype and comparable maternal and paternal impacts, while sociodemographics besides gender were not associated with parental QoL. The findings highlight the need for psychosocial interventions tailored to the Saudi cultural context to improve parental coping and QoL in families managing pediatric ADHD, which can facilitate more positive child outcomes.

## Appendices

Questionnaire

Demographics section

1.       Who fills out the questionnaire? (mother, father)

2.       Marital status of parents (married, divorced, widower)

3.       Is there a family history of any mental illness being diagnosed? (Yes\No)

4.       If there is a family history of any mental illness, was adequate healthcare sought? (Yes\No\Other)

5.      Does one of the parents suffer from any chronic medical conditions (diseases) (that require waiting, time, and a lot of effort, such as diabetes, high blood pressure, heart disease, etc.)? (Yes\No)

6.       Number of children in the family?

7.       Have any of your children ever been diagnosed with ADHD? (Yes\No)

8.       If you have a child with the disorder, do you have other children with the same disorder? (Yes\No)

9.       Have any of your children ever been diagnosed with another psychological disorder (other than ADHD) (Yes\No)

10.    How old is your child who has the disorder or who you think has the disorder?

11.    How old was your child when you suspect the onset of ADHD symptoms (write the year as a number) or write zero (0) if there are no symptoms)

12.    In your opinion, the time period required to treat a child diagnosed with ADHD:

Less than one year

One to two years

More than two years

13.    Gender of the child who has the disorder/or who you think has the disorder:

Male

Female

14.    Age of the mother

15.   Maternal education (college or above, high school or below)

16.   Employment status (employed, unemployed, retired)

17.   Age of the father

18.   Parental education (college or above, high school or below)

19.   Employment status (employed, unemployed, retired)

20.   Residence (rural, urban)

21.   Income status of the family (below 5,000, from 5,000 to 10,000, from 10,000 to 15,000, from 15,000 to 20,000, above 20,000)

22.   Is the family income sufficient for the parents’ life requirements? (Yes\No)

23.    Do you think that the quality of your life has had a positive impact on your child? (Yes\No)

NICHQ Vanderbilt Assessment Scale-PARENT Informant (used for diagnosing ADHD) section

1.     Does not pay attention to details or makes careless mistakes with, for example, homework?

Never

Occasionally

Often

Very often

2.    Has difficulty in keeping attention to what needs to be done?

Never

Occasionally

Often

Very often

3.    Does not seem to listen when spoken to directly?

Never

Occasionally

Often

Very often

4.    Does not follow through when given directions and fails to finish activities (not due to refusal or failure to understand)?

Never

Occasionally

Often

Very often

5.     Has difficulty organizing tasks and activities?

Never

Occasionally

Often

Very often

6.      Avoids, dislikes, or does not want to start tasks that require ongoing mental effort?

Never

Occasionally

Often

Very often

7.      Loses things necessary for tasks or activities (toys, assignments, pencils, or books)?

Never

Occasionally

Often

Very often

8.      Is easily distracted by noises or other stimuli?

Never

Occasionally

Often

Very often

9.     Is forgetful in daily activities (changing his clothes before bed, brushing his teeth, organizing his backpack)?

Never

Occasionally

Often

Very often

10.   Fidgets with hands or feet or squirms in the seat?

Never

Occasionally

Often

Very often

11.    Leaves the seat when remaining seated is expected (during meals, family gatherings on TV shows)?

Never

Occasionally

Often

Very often

12.     Runs about or climbs too much when remaining seated is expected

Never

Occasionally

Often

Very often

13.      Has difficulty playing or beginning quiet play activities?

Never

Occasionally

Often

Very often

14.      Is “on the go” or often acts as if “driven by a motor” Talks too much?

Never

Occasionally

Often

Very often

15.     Talks too much?

Never

Occasionally

Often

Very often

16.     Blurts out answers before questions have been completed

Never

Occasionally

Often

Very often

17.       Has difficulty waiting his or her turn?

Never

Occasionally

Often

Very often

18.       Interrupts or intrudes on others’ conversations and/or activities

Never

Occasionally

Often

Very often

19.     Argues with adults

Never

Occasionally

Often

Very often

20.    Loses temper (easily gets angry for things that don’t require it)

Never

Occasionally

Often

Very often

21.      Actively defies or refuses to go along with adults’ requests or rules

Never

Occasionally

Often

Very often

22.    Deliberately annoys people

Never

Occasionally

Often

Very often

23.    Blames others for his or her mistakes or misbehaviors

Never

Occasionally

Often

Very often

24.    Is touchy or easily annoyed by others

Never

Occasionally

Often

Very often

25.    Is angry or resentful

Never

Occasionally

Often

Very often

26.    Is spiteful and wants to get even

Never

Occasionally

Often

Very often

27.    Bullies, threatens, or intimidates others

Never

Occasionally

Often

Very often

28.    Starts physical fights

Never

Occasionally

Often

Very often

29.    Lies to get out of trouble or to avoid obligations (i.e., “cons” others)

Never

Occasionally

Often

Very often

30.    Is truant from school (skips school) without permission

Never

Occasionally

Often

Very often

31.    Is physically cruel to people

Never

Occasionally

Often

Very often

32.    Has stolen things that have value

Never

Occasionally

Often

Very often

33.    Deliberately destroys others’ property

Never

Occasionally

Often

Very often

34.    Has used a weapon that can cause serious harm (bat, knife, brick, gun)

Never

Occasionally

Often

Very often

35.   Is physically cruel to animals

Never

Occasionally

Often

Very often

36.    Has deliberately set fires to cause damage

Never

Occasionally

Often

Very often

37.    Has broken into someone else’s home, business, or car

Never

Occasionally

Often

Very often

38.    Has stayed out at night without permission

Never

Occasionally

Often

Very often

39.    Has run away from home overnight

Never

Occasionally

Often

Very often

40.    Has forced someone into sexual activity

Never

Occasionally

Often

Very often

41.    Is fearful, anxious, or worried

Never

Occasionally

Often

Very often

42.   Is afraid to try new things for fear of making mistakes

Never

Occasionally

Often

Very often

43.    Feels worthless or inferior

Never

Occasionally

Often

Very often

44.    Blames self for problems, feels guilty

Never

Occasionally

Often

Very often

45.    Feels lonely, unwanted, or unloved; complains that “no one loves him or her”

Never

Occasionally

Often

Very often

46.    Is sad, unhappy, or depressed

Never

Occasionally

Often

Very often

47.    Is self-conscious or easily embarrassed

Never

Occasionally

Often

Very often

48.   Overall school performance

Excellent

Above average

Average

Somewhat of a problem

Problematic

49.    Reading

Excellent

Above average

Average

Somewhat of a problem

Problematic

50.    Writing

Excellent

Above average

Average

Somewhat of a problem

Problematic

51.    Mathematics

Excellent

Above average

Average

Somewhat of a problem

Problematic

52.    Relationship with parents

Excellent

Above average

Average

Somewhat of a problem

Problematic

53.    Relationship with siblings

Excellent

Above average

Average

Somewhat of a problem

Problematic

54.    Relationship with peers

Excellent

Above average

Average

Somewhat of a problem

Problematic

55.    Participation in organized activities (e.g., teams)

Excellent

Above average

Average

Somewhat of a problem

Problematic

Multicultural Quality of Life Index section

1. Physical well-being (feeling energetic, free of pain and physical problems)

Poor 1 2 3 4 5 6 7 8 9 10 Excellent

2. Psychological/emotional well-being (feeling good and comfortable with yourself)

Poor 1 2 3 4 5 6 7 8 9 10 Excellent

3. Self-care and independent functioning (carrying out daily living tasks; making own decisions)

Poor 1 2 3 4 5 6 7 8 9 10 Excellent

4. Occupational functioning (able to carry out work, school, and homemaking duties)

Poor 1 2 3 4 5 6 7 8 9 10 Excellent

5. Interpersonal functioning (able to respond and relate well to family, friends, and groups)

Poor 1 2 3 4 5 6 7 8 9 10 Excellent

6. Social-emotional support (availability of people you can trust and who can offer help and

emotional support)

Poor 1 2 3 4 5 6 7 8 9 10 Excellent

7. Community and services support (pleasant and safe neighborhood, access to financial,

informational and other resources)

Poor 1 2 3 4 5 6 7 8 9 10 Excellent

8. Personal fulfillment (experiencing a sense of balance, dignity, and solidarity; enjoying sexuality,

the arts, etc.)

Poor 1 2 3 4 5 6 7 8 9 10 Excellent

9. Spiritual fulfillment (experiencing faith, religiousness, and transcendence beyond ordinary

material life)

Poor 1 2 3 4 5 6 7 8 9 10 Excellent

10. Global perception of quality of life (feeling satisfied and happy with your life in general)

Poor 1 2 3 4 5 6 7 8 9 10 Excellent
